# Immunological Control of HIV-1 Disease Progression by Rare Protective HLA Allele

**DOI:** 10.1128/jvi.01248-22

**Published:** 2022-11-03

**Authors:** Yu Zhang, Takayuki Chikata, Nozomi Kuse, Hayato Murakoshi, Hiroyuki Gatanaga, Shinichi Oka, Masafumi Takiguchi

**Affiliations:** a Division of International Collaboration Research and Tokyo Joint Laboratory, Joint Research Center for Human Retrovirus Infection, Kumamoto University, Kumamoto, Japan; b AIDS Clinical Center, National Center for Global Health and Medicine, Tokyo, Japan; Emory University

**Keywords:** HIV-1, CD8^+^ T cells, HLA-B*67:01, protective epitope

## Abstract

Rare HLA alleles such as HLA-B57 are associated with slow progression to AIDS. However, the evidence for the advantage of rare protective alleles is limited, and the mechanism is still unclear. Although the prevalence of HLA-B*67:01 is only 1.2% in Japan, HLA-B*67:01-positive (HLA-B*67:01^+^) individuals had the lowest plasma viral load (pVL) and highest CD4 count in HIV-1 clade B-infected Japanese individuals. We investigated the mechanism of immunological control of HIV-1 by a rare protective allele, HLA-B*67:01. We identified six novel HLA-B*67:01-restricted epitopes and found that T cells specific for four epitopes were significantly associated with good clinical outcomes, pVL and/or CD4 count. The wild type or cross-reactive sequences of three protective and immunodominant Pol and Gag epitopes were found in around 95% of the circulating HIV-1, indicating that T cells specific for three conserved or cross-reactive epitopes contributed to good clinical outcomes. One escape mutation (Nef71K) in the Nef protective epitope, which was selected by T cells restricted by either HLA allele in the HLA-B*67:01-C*07:02 haplotype, affected the HLA-B*67:01-restricted RY11-specific T-cell recognition. These results imply that the further accumulation of the Nef71K mutation in the population will negatively affect the control of HIV-1 replication by RY11-specific CD8^+^ T cells in HIV-1-infected HLA-B*67:01^+^ individuals. The present study demonstrated that conserved or cross-reactive epitope-specific T cells mainly contribute to control of HIV-1 by a rare protective allele, HLA-B*67:01.

**IMPORTANCE** HLA-B57 is a relatively rare allele around world and the strongest protective HLA allele in Caucasians and African black individuals infected with HIV-1. Previous studies suggested that the advantage of this allele in HIV-1 disease progression is due to a strong functional ability of HLA-B57-restricted Gag-specific T cells and lower fitness of mutant viruses selected by the T cells. HLA-B*57 is a very rare allele and has not been reported as a protective allele in Asian countries, whereas a rare allele, HLA-B*67:01, was shown to be a protective allele in Japan. Therefore, the analysis of HLA-B*67:01-restricted T cells is important to clarify the mechanism of immunological control of HIV-1 by a rare protective HLA allele in Asia. We found that HLA-B*67:01-restricted T cells specific for three conserved or cross-reactive Gag and Pol epitopes are associated with good clinical outcomes in HLA-B*67:01^+^ individuals. It is expected that T cells specific for conserved or cross-reactive epitopes contribute to a curing treatment.

## INTRODUCTION

Several human leukocyte antigen (HLA) class I alleles or haplotypes are associated with human immunodeficiency virus type 1 (HIV-1) disease progression to AIDS and/or with clinical outcomes (1–9). Large cohort studies of HIV-1-infected Caucasian and African populations have shown that HLA-B*57 and HLA-B*27 alleles have a protective effect against the progression of the disease (1, 3, 6, 10–16). Genome-wide association studies have further demonstrated strong genetic associations between single-nucleotide polymorphisms within HLA-B*57 and HLA-B*52 and their protective effect against HIV-1 disease progression (7, 14, 17, 18). These studies implied that HIV-1-specific T cells restricted by these protective HLA alleles contribute to suppression of HIV-1 replication in HIV-1-infected individuals. Indeed, previous studies have demonstrated that the induction of HLA-B*57- and HLA-B*27-restricted CD8^+^ T cells targeting HIV-1 Gag epitopes was associated with lower plasma viral load (pVL) in HLA-B*57-positive (HLA-B*57^+^) and HLA-B*27^+^ individuals, respectively (19–23). These findings suggest that Gag epitope-specific T cells have an important role in suppression of HIV-1 replication in individuals with the protective HLA alleles. HLA-B*57 is a very rare allele in Asian countries, including Japan, Thailand, and northern China (http://www.allelefrequencies.net) and was not reported as a protective allele in Asian countries, suggesting that HIV-1 replication may be controlled by other protective HLA alleles in these countries. A previous study showed that three HLA alleles, HLA-B*67:01, HLA-B*52:01, and HLA-C*12:02, were significantly associated with both low pVL and a high CD4 count in HIV-1 subtype B-infected Japanese individuals, indicating that these three HLA alleles have a protective effect on HIV-1-infected Japanese individuals (8). Furthermore, it has been demonstrated that the epitope-specific CD8^+^ T cells restricted by these three protective HLA alleles effectively suppressed HIV-1 replication in Japanese individuals (24–26).

HLA-B*67:01 is a rare allele in the world. It is absent or very rare (<0.1%) in Caucasians and Africans, whereas its prevalence in some Asian populations is 0.9%–1.2% (http://www.allelefrequencies.net). Although its prevalence is only 1.2% in Japan (http://hla.or.jp), HLA-B*67:01^+^ individuals had the lowest pVL and highest CD4 count in HIV-1 clade B-infected Japanese individuals (8). The protective effect of this allele was also reported in HIV-1-infected Chinese people in northern China (27). A previous study on 15 HIV-1-infected HLA-B*67:01^+^ Japanese individuals identified only two Gag epitopes restricted by HLA-B*67:01 and suggested that these Gag-specific T cells reduced the pVL (24). HLA-B*67:01-restricted T cells specific for other proteins have not been identified; hence, the role of HLA-B*67:01-restricted HIV-1-specific T cells in the slow progression of the disease remains only partially clarified.

A previous study on Caucasian and African-American populations showed an advantage of rare HLA alleles such as the HLA-B*58 supertype, which includes HLA-B*57, on HIV-1 disease progression (5). The advantage of HLA-B*57 may be explained by a strong ability of Gag-specific HLA-B*57-restricted T cells to suppress HIV-1 replication (11, 28–30) and lower the fitness of mutant viruses selected by these T cells (30–32). It was speculated that the effect of preadapted HIV-1 transmission was minimal in individuals harboring rare HLA alleles (33). To further understand the mechanism of the immunological control of HIV-1 by rare protective HLA alleles, it is valuable to investigate the role of HIV-1-specific T cells restricted by other HLA alleles which are less prevalent than HLA-B*57. The effect of preadapted HIV-1 transmission on such very rare alleles may be minimal compared with HLA-B*57. HLA-B*67:01 is a good target allele for these studies because the frequency of this allele is approximately 5 times lower than that of HLA-B*57.

In the present study, we investigated the HLA-B*67:01-mediated control of HIV-1 disease progression. We collected samples from 24 treatment-naive HIV-1 subtype B-infected HLA-B*67:01^+^ individuals and then sought to identify novel HLA-B*67:01-restricted HIV-1 epitopes. We further analyzed the role of T cells specific for these HLA-B*67:01-restricted epitopes in HIV-1 suppression and accumulation of mutations in these epitopes. Here, we clarified the role of the very rare allele HLA-B*67:01 in HIV-1 infection.

## RESULTS

### T-cell responses to cocktails of overlapping 11-mer peptides in 15 HLA-B*67:01^+^ individuals chronically infected with HIV-1 subtype B.

**FIG 1:**
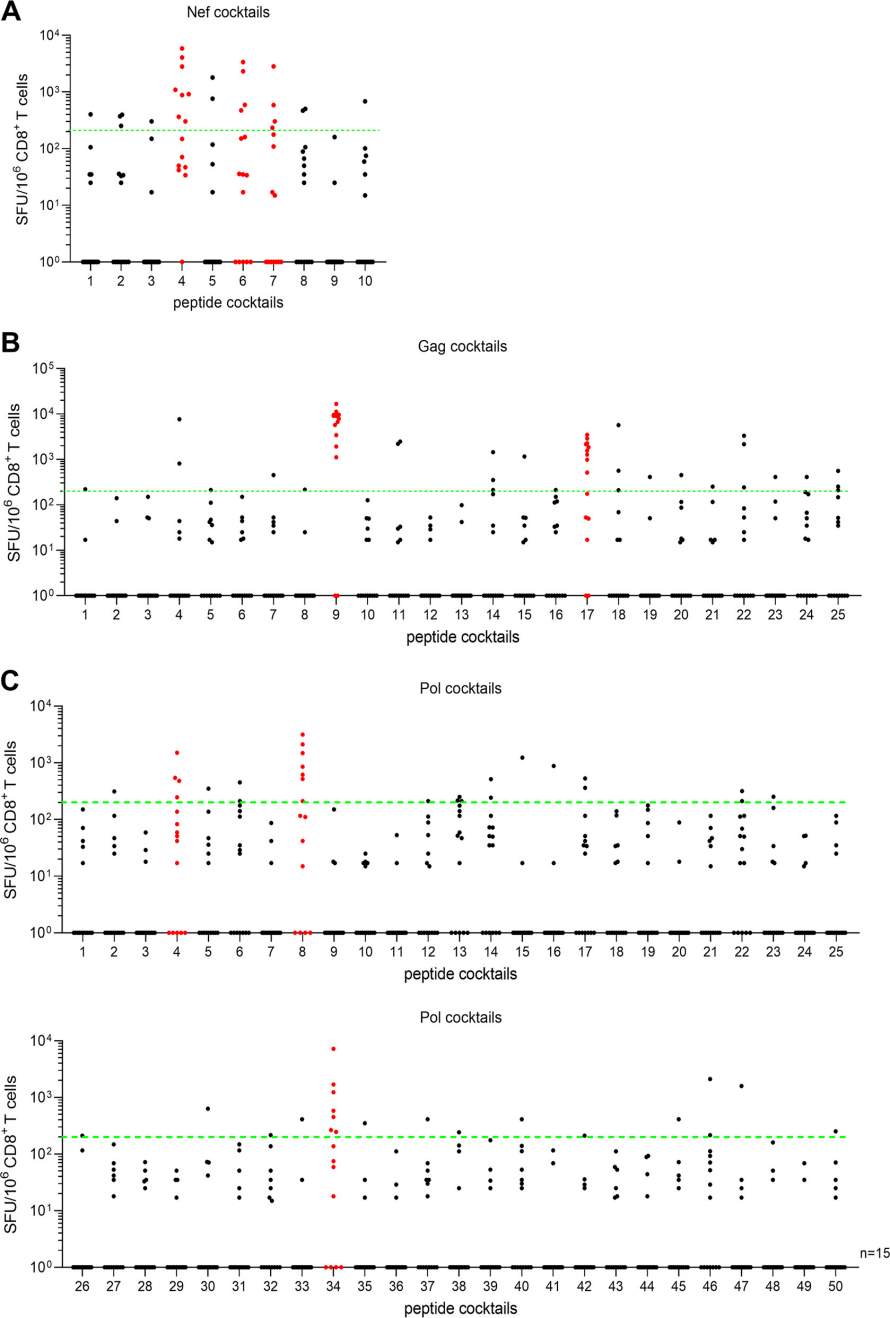
T-cell responses to peptide cocktails containing overlapping 11-mer HIV-1 clade B-derived peptides spanning Nef, Gag, and Pol regions. (A to C) T-cell responses to Nef (A), Gag (B), and Pol (C) peptide cocktails were analyzed by performing an *ex vivo* IFN-γ ELISpot assay using PBMCs from 15 treatment-naive HLA-B*67:01^+^ HIV-1-infected Japanese individuals. Each peptide cocktail includes 8 to 10 11-mer overlapping peptides. T-cell responses to the peptide cocktail at a concentration of 1 μM were measured. The dotted line at 200 spots/10^6^ CD8^+^ T cells indicates the threshold for a positive response.

To identify novel HLA-B*67:01-restricted immunodominant epitopes, we first analyzed the responses of peripheral blood mononuclear cells (PBMCs) from 15 HLA-B*67:01^+^ individuals chronically infected with HIV-1 subtype B to 85 cocktails (10 Nef, 25 Gag, and 50 Pol cocktails) of overlapping 11-mer peptides spanning consensus sequences of Nef, Gag, and Pol of HIV-1 clade B by performing a interferon gamma (IFN-γ) ELISpot assay. Each peptide cocktail included 10 11-mer peptides. We found positive T-cell responses to three Nef (Nef4, 6, and 7), two Gag (Gag9 and 17), and three Pol (Pol4, 8, and 34) cocktails in at least four individuals (responder frequency, >25%), suggesting that these peptide cocktails may include HLA-B*67:01-restricted immunodominant epitopes (Fig. 1). Indeed, two Gag cocktails, Gag9 and Gag17, included the previously reported HLA-B*67:01-restricted epitopes, GagTL9, GagEM11, and GagNL11 (24, 34).

### Identification of novel HLA-B*67:01-restrcited HIV-1 T-cell epitopes.

**FIG 2:**
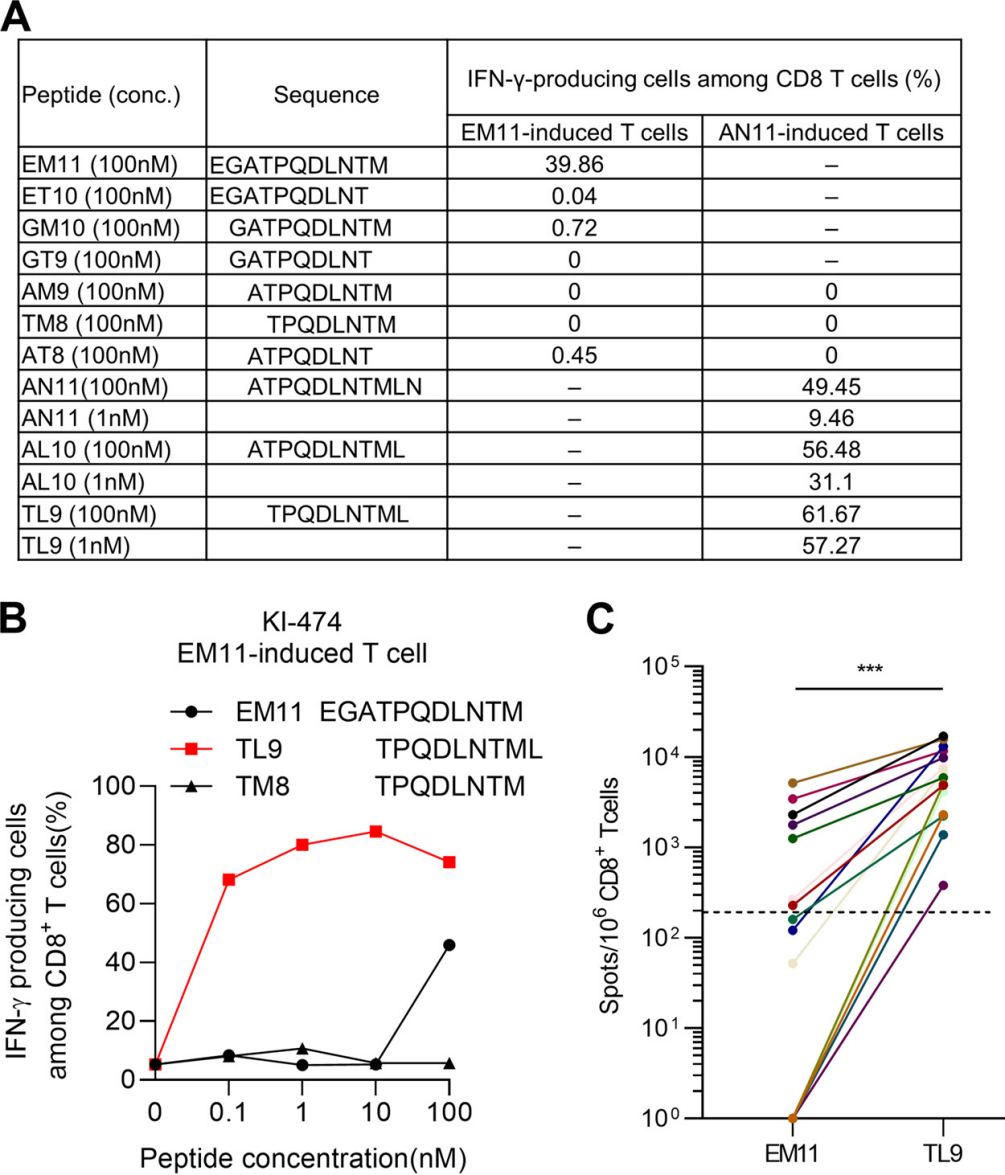
Recognition of truncated GagEM11 and GagAN11 peptides by EM11-induced and AN11-induced T cells. (A) Recognition of truncated GagEM11 and GagAN11 peptides by EM11-induced and AN11-induced T cells. EM11-induced and AN11-induced T cells were induced by stimulating PBMCs from HIV-1-infected HLA-B*67:01^+^ individual KI-474 with GagEM11 and GagAN11 peptides, respectively. Recognition of the truncated peptides by these T cells was analyzed by performing an ICS assay. The result of this analysis was partially shown as supplemental data in a previous study (24). (B) Recognition of EM11, TL9, and TM8 peptides by EM11-induced T cells. T-cell responses to 721.221-B*67:01 cells prepulsed with EM11, TL9, or TM8 peptide were analyzed by ICS assay. (C) T-cell responses to EM11 and TL9 peptides in 15 HIV-1-infected HLA-B*67:01 individuals were analyzed by performing an ELISpot assay. The dotted line at 200 spots/10^6^ CD8^+^ T cells indicates a threshold for a positive response. Statistical analysis was conducted by use of a paired *t* test. ***, *P* < 0.001.

Our previous study showed that GagEM11 (EGATPQDLNTM)-induced T cells did not recognize the truncated peptides, whereas GagAN11 (ATPQDLNTMLN)-induced T cells recognized GagTL9 (TPQDLNTML) to a greater extent than the AL10 and AN11 peptides (24). From these results, we previously concluded that EM11 and TL9 are HLA-B*67:01 epitopes. We summarize the previous data related to the recognition of EM11-induced and AN11-induced T cells for the truncated peptides of GagEM11 and Gag AN11 (Fig. 2A). However, because GagEM11 (EGATPQDLNTM) and GagTL9 (TPQDLNTML) overlap, we speculated that these peptides are cross-recognized or that another peptide is the epitope. We therefore investigated the recognition of EM11-induced T-cells established from KI-474 for the EM11, TL9, and TM8 peptides at different concentrations by performing an intracellular cytokine staining (ICS) assay (Fig. 2B). These T cells recognized EM11 and TL9 peptides at a concentration of 100 nM, whereas they recognized only TL9 peptide at 0.1 to 10 nM. These results suggested that EM11 is not an HLA-B*67:01-restricted epitope. We analyzed T-cell responses to EM11 and TL9 peptides further in 15 HIV-1-infected HLA-B*67:01^+^ individuals by performing an *ex vivo* ELISpot assay and compared the T-cell responses to these peptides. All responders to TL9 showed weaker or no responses to EM11 at a concentration of 100 nM (Fig. 2C). These results suggest that the responses of EM11-induced T cells to the EM11 peptide may be related to the cross-recognition of the overlapping part of the EM11 peptide by the T-cell receptor (TCR) of TL9-specific T cells. These findings indicate that TL9 is an immunodominant epitope and EM11 is not an epitope.

**FIG 3:**
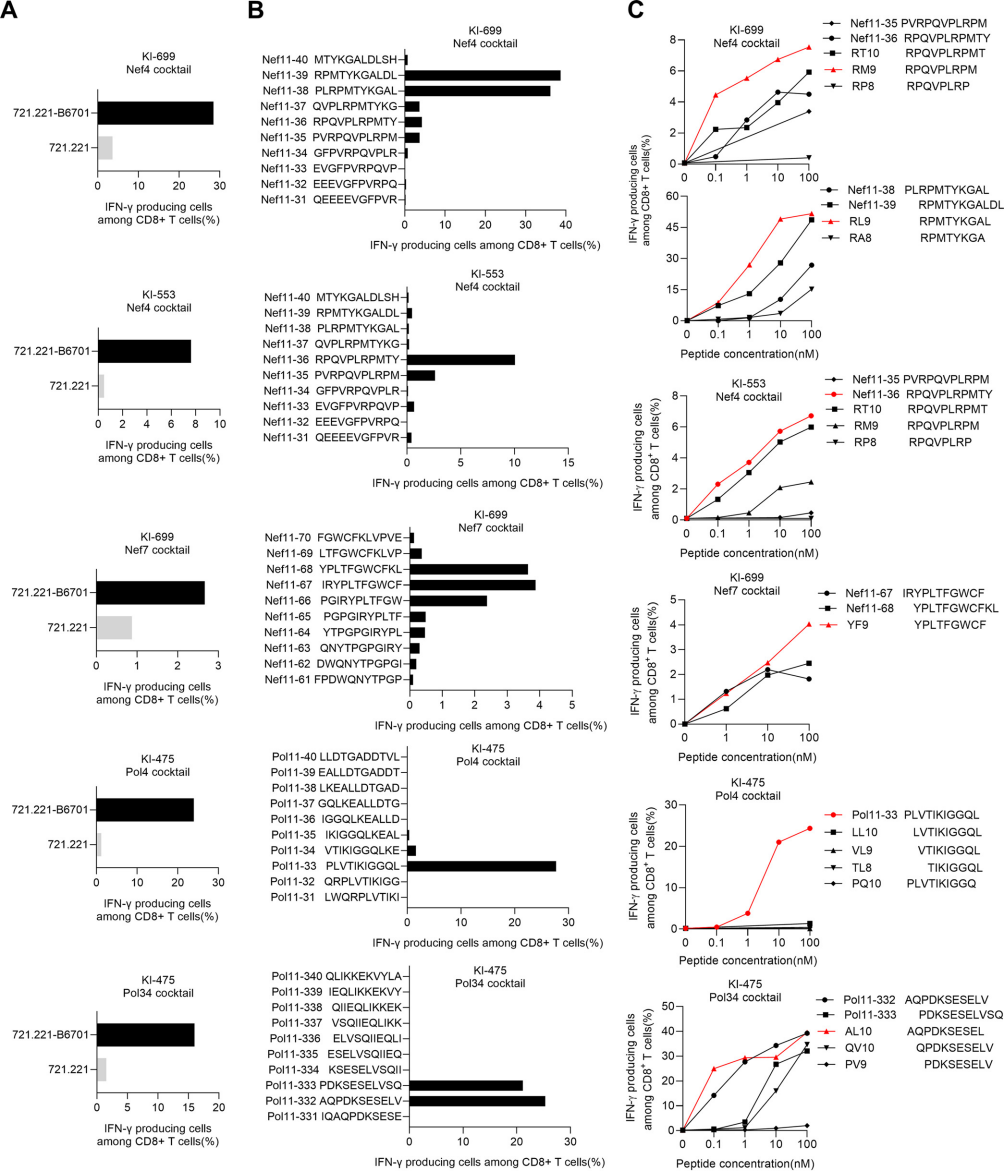
Identification of novel HLA-B*67:01-restricted T-cell epitopes. (A) Analysis of HLA-B*67:01-restricted T-cell responses to Nef4, Nef7, Pol4, and Pol34 peptide cocktails in 3 individuals, KI-699, KI-553, and KI-475, who showed positive responses to the corresponding peptide cocktails in the *ex vivo* ELISpot assay. Responses of the peptide cocktail-induced T cells to 721.221-B*67:01 cells or 721.221 cells pulsed with the peptide cocktail at 1 μM were analyzed by ICS assay. (B and C) Identification of HLA-B*67:01-restricted optimal epitope peptides. Responses of the peptide cocktail-induced bulk T cells to the 721.221-B*67:01 cells pulsed with overlapping 11-mer peptides at 1 μM (B) and those with truncated ones from 0.1 to 100 nM (C) were analyzed using ICS assay.

Next, we analyzed three Nef (Nef4, 6, and 7) and three Pol (Pol4, 8, and 34) peptide cocktails to identify novel HLA-B*67:01-restricted T-cell epitopes. To elucidate HLA-B*67:01-restriction of T-cell responses to these Nef and Pol cocktails, we selected seven responders to these cocktails in the ELISpot assay (Table 1). We stimulated PBMCs from these responders with each peptide cocktail and cultured them for approximately 2 weeks. HLA-B*67:01-restricted T-cell responses to each peptide cocktail in the cultured cells were then analyzed by performing the ICS assay. HLA-B*67:01-restricted T-cell responses to four cocktail peptides were found in these responders as follows: T-cell responses to Nef4 in individuals KI-699 and KI-553, to Nef7 in individual KI-699, to Pol4 in individual KI-475, and to Pol34 in individual KI-475 (Fig. 3A).

**TABLE 1:** T-cell responses to 8 peptide cocktails in 7 HLA-B*67:01^+^ responders^*a*^^,^^*b*^

ID	HLA-A		HLA-B		HLA-C		T cell response to cocktails (spots/10^6^ CD8^+^ T cells)							
							Nef4	Nef6	Nef7	Pol4	Pol8	Pol34	Gag9	Gag17
KI-699	A*02:01	A*02:01	B*15:01	B*67:01	C*03:03	C*07:02	**5875**	160	**586**	**479**	106	**266**	**5,751**	**1,278**
KI-475	A*11:01	A*24:02	B*52:01	B*67:01	C*07:02	C*12:02	71	35	0	**247**	**1,485**	**1,697**	**6,717**	**1,555**
KI-690	A*11:01	A*24:02	B*52:01	B*67:01	C*07:02	C*12:02	149	**3,368**	0	**545**	0	**1,238**	**3,417**	50
KI-553	A*11:01	A*11:01	B*40:01	B*67:01	C*03:04	C*07:02	**882**	**2,309**	15	**1,500**	15	59	**9,427**	176
KI-794	A*24:02	A*26:01	B*07:02	B*67:01	C*07:02	C*07:02	34	34	0	138	**3,062**	0	**11,249**	**2,890**
KI-474	A*11:01	A*24:02	B*40:06	B*67:01	C*07:02	C*08:01	**1,090**	0	17	17	**519**	138	**9,138**	17
KI-855	A*02:01	A*24:02	B*52:01	B*67:01	C*07:02	C*12:02	**364**	**473**	109	0	**618**	0	**16,730**	**1,855**

a Responses of PBMCs from 7 HIV-1-infected HLA-B*67:01^+^ individuals to 8 cocktails of overlapping 11-mer peptides at a concentration of 1 μM were analyzed by performing an *ex vivo* IFN-γ ELISpot assay.

b Bold indicates the positive responses (>200 spots) in the ELISpot assay.

We then attempted to identify the optimal epitopes using overlapping 11-mer peptides and their truncated peptides. We found that Nef4-induced CD8^+^ T-cells derived from individual KI-699 strongly recognized Nef11-38/39 and weakly recognized Nef11-35/36/37 single peptides, while Nef7-induced T-cells from the same individual recognized Nef11-66/67/68 single peptides (Fig. 3B). These results suggest that Nef4 and Nef7 cocktails may include at least two HLA-B*67:01-restricted epitopes. We further analyzed T-cell recognition of truncated peptides covering overlapping parts of these single peptides. Nef4-induced T cells from individual KI-699 recognized RM9 (RPQVPLRPM) and RL9 (RPMTYKGAL) peptides more than other truncated peptides, while those from individual KI-553 recognized Nef11-36 (RY11: RPQVPLRPMTY) more than the truncated peptides (Fig. 3C). These results indicated that RM9, RL9, and RY11 are optimal epitopes, though it remains possible that RT10 is also presented as a superimposed epitope with RY11. Furthermore, Nef7-induced T cells recognized YF9 (YPLTFGWCF) more than two 11-mer peptides (Fig. 3C). Taken together, these results indicate that RM9, RL9, YF9, and RY11 are HLA-B*67:01-restricted Nef epitopes.

Pol4-induced and Pol34-induced CD8^+^ T cells responded to Pol11-33 and Pol11-332/333 peptides, respectively (Fig. 3B). To identify HLA-B*67:01-restricted epitopes, we analyzed T-cell recognition of Pol11-33 truncated peptides or of sequences covering overlapping parts of Pol11-332 and Pol11-333 peptides. Pol4-induced CD8^+^ T cells recognized an 11-mer peptide (Pol PL11: PLVTIKIGGQL), but not any truncated peptides (Fig. 3C), suggesting that PL11 is an epitope. Pol34-induced CD8^+^ T cells recognized Pol AL10 (AQPDKSESEL) and Pol11-332 (AV11) more than Pol11-333 or other truncated peptides (Fig. 3C). Although it remains possible that both AL10 and AV11 are epitopes, we determined that AL10 is the optimal epitope because the T cells recognized AL10 more than AV11 at 0.1 nM these peptides.

**FIG 4:**
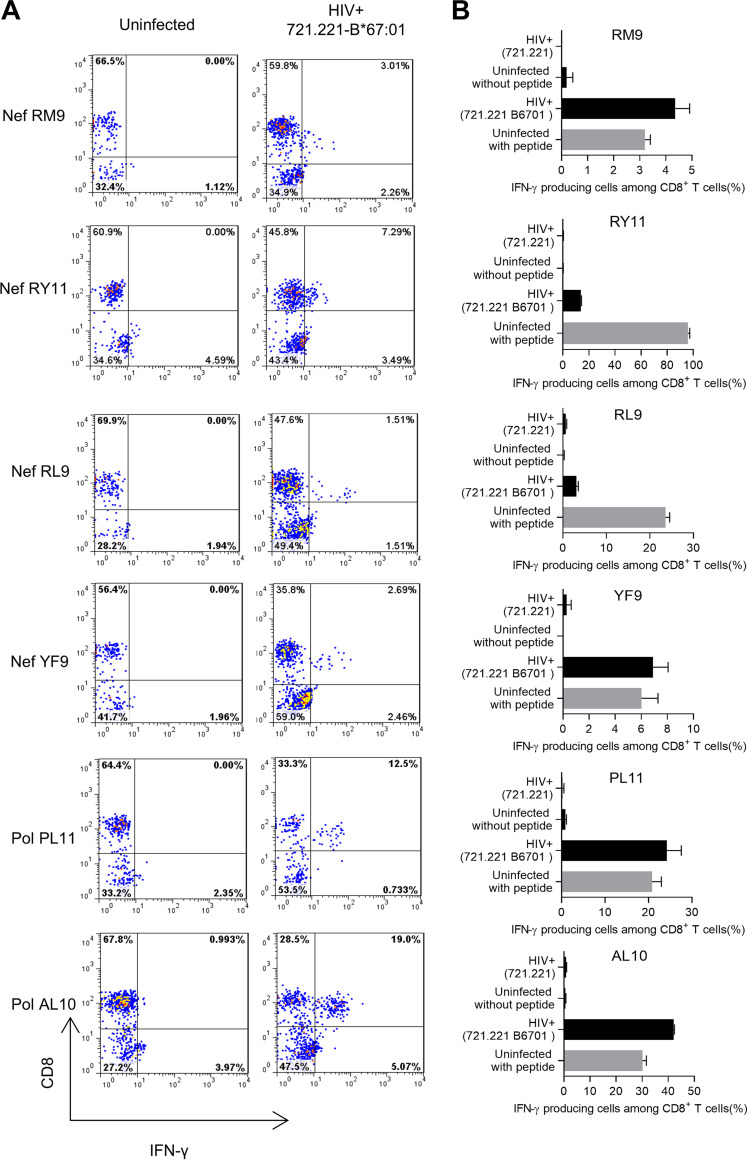
Recognition of HIV-1-infected cells by HLA-B*67:01-restricted HIV-1-specific T cells. Responses of T cells specific for the NefRM9, RY11, RL9, or YF9 epitope to 721.221-B*67:01 cells infected with NL4-3_SF2Nef_ and those of T cells specific for the Pol PL11 or AL10 epitope to 721.221-B*67:01 cells infected with NL4-3 were analyzed by performing the ICS assay. The frequencies of p24 antigen-positive cells among 721.221-B*67:01 cells infected with NL4-3_SF2Nef_ or NL4-3 were 51.0% or 34.4%, respectively, and those of 721.221 infected with NL4-3_SF2Nef_ or NL4-3 were 60.0% and 36.3%, respectively. The responses of the T cells to 721.221-B*67:01 cells prepulsed with epitope peptides at 100 nM were used as a positive control. (A) Representative results of each epitope-specific T cell are shown. (B) The frequency of the IFN-γ^+^ cells among CD8^+^ T cell population. The data are presented as the mean and standard deviation (SD) (*n* = 3).

Next, we investigated recognition of HIV-1-infected cells by CD8^+^ T cells specific for these novel epitope peptides. Most of the CD8^+^ T cells induced by NefRM9 and NefYF9 peptides effectively recognized HIV-1-infected 721.221-B*67:01 cells, while those specific for Nef RL9 and Nef RY11 responded to HIV-1-infected 721.221-B*67:01 cells weakly but significantly (Fig. 4A and B). T cells induced by PolPL11 or PolAL10 peptide effectively recognized HIV-1-infected 721.221-B*67:01 cells (Fig. 4A and B). These results indicate that these six peptides are presented as naturally occurring peptides in HIV-1-infected cells.

### T-cell responses to eight HLA-B*67:01-restricted epitopes and their association with clinical outcomes.

**FIG 5:**
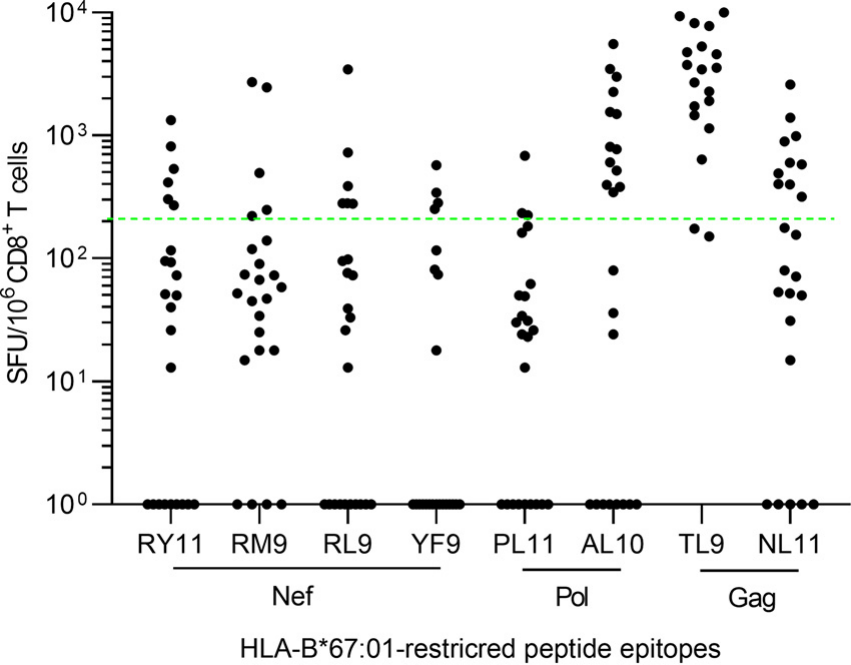
T-cell responses to 8 HLA-B*67:01-restricted epitopes in chronically HIV-1-infected HLA-B*67:01^+^ individuals. T-cell responses to 8 HLA-B*67:01-restricted epitope peptides at a concentration of 100 nM were analyzed by an *ex vivo* IFN-γ ELISpot assay in PBMCs from 24 treatment-naive HLA-B*67:01^+^ individuals chronically infected with HIV-1. The dotted line at 200 spots/10^6^ CD8^+^ T cells indicates the threshold for a positive response.

To investigate the frequency of HIV-1-infected individuals harboring T cells specific for eight HLA-B*67:01-restricted epitopes, we analyzed 24 HIV-1-infected HLA-B*67:01^+^ individuals by performing the *ex vivo* ELISpot assay. The number of responders to NefRY11, NefRM9, NefRL9, and NefYF9 was 6, 5, 6, and 4, respectively, whereas that to PolPL10 and Pol AL10 was 3 and 13, respectively (Fig. 5). The T cells specific for GagTL9 and GagNL11 were detected in 22 and 10 HLA-B*67:01^+^ individuals, respectively (Fig. 5). The T cells specific for PolAL10, GagTL9, and GagNL11 were detected in more than 40% of the 24 HLA-B*67:01^+^ individuals tested, indicating that they were the immunodominant epitopes.

**FIG 6:**
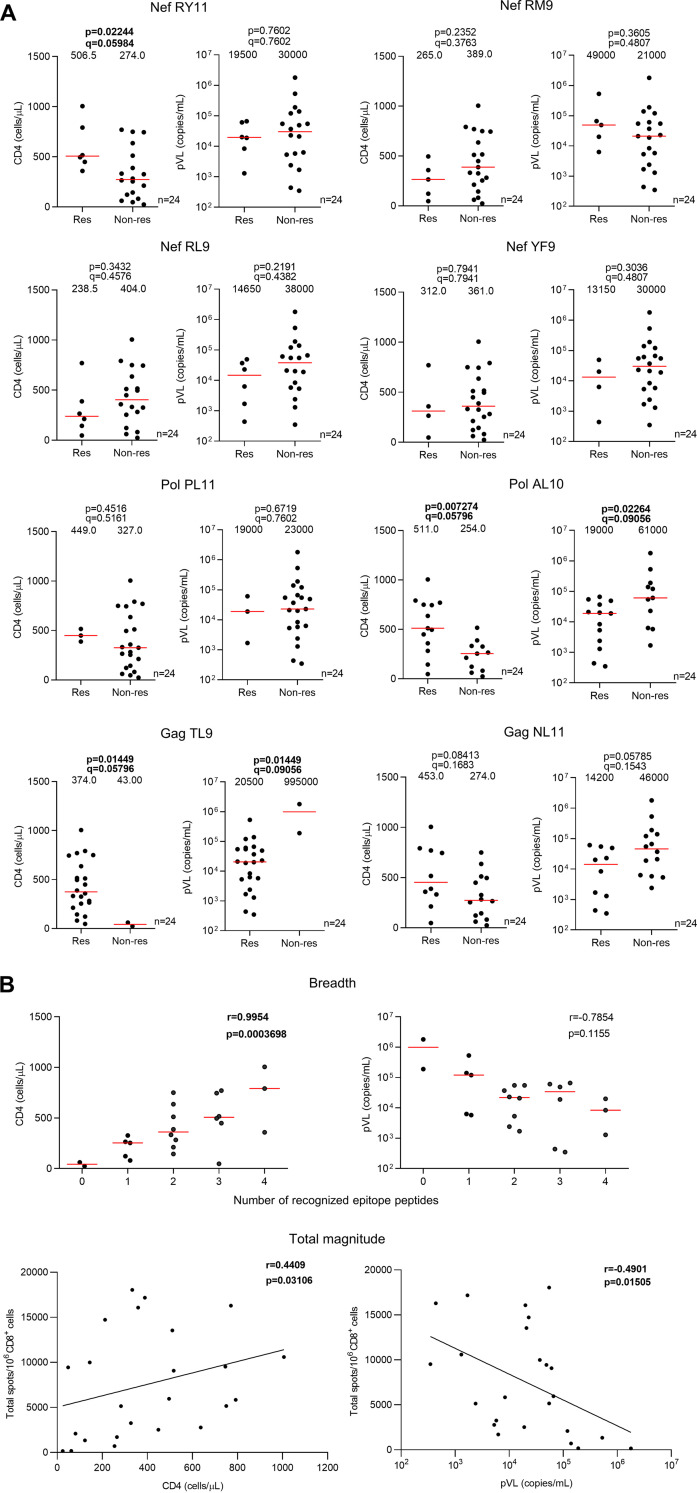
Correlation of T-cell responses to HLA-B*67:01-restricted epitopes with pVL and CD4 count. (A) T-cell responses to 4 Nef (RY11, RM9, RL9, and YF9), 2 Pol (PL11 and AL10), and 2 Gag (TL9 and NL11) epitope peptides were determined by an *ex vivo* IFN-γ ELISpot assay in 24 treatment-naive HIV-1-infected HLA-B*67:01^+^ individuals. Differences in pVL and CD4 count between responders (Res) and nonresponders (Non-res) were statistically analyzed using the Mann-Whitney test. Multiple tests were performed using the *q* value, a measure of significance in terms of the false-discovery rate. The value in each graph represents the median of pVL and CD4 counts. (B) Correlations of total breadth and magnitude of T-cell responses to 4 protective epitope peptides (Nef RY11, Pol AL10, Gag TL9, and Gag NL11) with pVL and CD4 count were statistically analyzed using Pearson’s correlation coefficient test and the Spearman rank correlation test, respectively.

We next investigated the effect of CD8^+^ T cells specific for these eight HLA-B*67:01-restricted epitopes on clinical outcomes of 838 Japanese individuals chronically infected with HIV-1 subtype B (24 HLA-B*67:01^+^ and 814 HLA-B*67:01^–^). The median pVL (copies/mL) and CD4 counts (cells/μL) of the 24 HLA-B*6701^+^ and 814 HLA-B*6701^–^ individuals were 22,000 (pVL)/332.5 (CD4 count) and 39,000 (pVL)/318 (CD4 count), respectively. Responders to PolAL10 or GagNL11 had significantly higher CD4 counts and lower pVL than the nonresponders, while responders to NefRY11 or GagTL9 had a significantly higher CD4 count or a significantly lower pVL than nonresponders, respectively (Table 2). We further analyzed the association of T-cell responses to these eight epitopes with pVL and CD4 counts among 24 HLA-B*67:01^+^ individuals. HLA-B*67:01^+^ responders to PolAL10 or GagTL9 had significantly lower pVL and higher CD4 counts than HLA-B*67:01^+^ nonresponders, while responders to NefRY11 had significantly higher CD4 counts than the nonresponders (Fig. 6A). Responders to GagNL11 showed a trend for a lower pVL and higher CD4 count than nonresponders (Fig. 6A). These results suggest that HLA-B*67:01-restricted T cells specific for NefRY11, PolAL10, GagTL9, or GagNL11 can suppress HIV-1 replication in HIV-1-infected individuals. The breadth and total magnitude of these four epitope-specific T-cell responses correlated positively with the CD4 count (breadth: *r *=* *0.99, *P = *3.7 × 10^−4^; total magnitude: *r *=* *0.44, *P = *0.031) and inversely with the pVL (total magnitude: *r* = −0.49, *P = *0.015; Fig. 6B). These findings indicate that the T cells specific for these four HLA-B*67:01-restricted epitopes together contribute to suppression of the disease progression.

**TABLE 2:** Association of T-cell responses to HLA-B*67:01-restricted epitope peptides with pVL or CD4^+^ T cell count^*a*^

Epitope	Sequence	Location	Frequency		Median pVL(copies/mL)		Median CD4 (cells/μL)		*P* value		*q* value	
			Res	Non-res	Res	Non-res	Res	Non-res	pVL	CD4	pVL	CD4
RY11	RPQVPLRPMTY	Nef 71–81	6	832	19,500	39,000	506.5	317.5	0.2499	**0.0032**	0.3332	**0.0140**
RM9	RPQVPLRPM	Nef 71–79	5	833	49,000	39,000	265	319	0.8486	0.4742	0.8486	0.6223
RL9	RPMTYKGAL	Nef 77–85	6	832	14,650	39,000	238.5	319	0.0601	0.5445	0.1202	0.6223
YF9	YPLTFGWCF	Nef 135–143	4	834	13,150	39,000	312	319	0.1148	0.9900	0.1837	0.9900
PL11	PLVTIKIGGQL	Pol 65–75	3	835	19,000	39,000	449	318	0.3506	0.1363	0.4007	0.2181
AL10	AQPDKSESEL	Pol 663–672	13	825	19,000	40,000	511	317	**0.0083**	**0.0035**	**0.0624**	**0.0140**
TL9	TPQDLNTML	Gag180–188	22	816	20,500	39,500	374	317.5	**0.0388**	0.0784	0.1035	0.1568
NL11	NPDCKTILKAL	Gag327–337	10	828	14,200	39,000	453	317.5	**0.0156**	**0.0372**	**0.0624**	**0.0992**

a The statistical analyses of differences in pVL or CD4 count between responders to each epitope and nonresponses were conducted by using the two-tailed Mann-Whitney test. Multiple tests were performed using the *q* value, a measure of significance in terms of the false-discovery rate. Significant differences (*P* < 0.05, *q* < 0.1) are indicated in bold.

### Effective recognition of circulating HIV-1 by T cells specific for Gag and Pol protective epitopes.

To clarify the variations in the four protective epitopes among circulating HIV-1, we identified the sequences corresponding to these epitopes in 321 to 351 HIV-1-infected Japanese individuals (Table 3). The wild-type (WT) sequences of GagTL9 and GagNL11 were highly conserved (>96%) among these individuals, while that of PolAL10 was detected in 83% of 348 individuals. The WT sequence of NefRY11 was found in 59.5% of 321 individuals. These results indicate that the Gag and Pol protective epitopes were relatively conserved among circulating viruses. Mutations in these two Gag epitopes were not found in HLA-B*67:01^+^ individuals. PolAL10-10I was found in 15.2% of 348 individuals, whereas it was found in 29.2% of 24 HLA-B*67:01^+^ individuals, implying that Pol672I is an HLA-B*67:01-associated mutation. However, there was no significant association of this mutant with HLA-B*67:01 (*P = *0.0709).

**TABLE 3:** Circulating HIV-1 sequences corresponding to 4 protective HLA-B*67:01-restricted epitopes

Epitope	Location	Sequence	Frequency in all individuals (%)	Frequency in HLA^+^ individuals (R)	Frequency in HLA^–^ individuals (%)
Nef RY11	Nef71-81	RPQVPLRPMTY	191/321 (59.5)	9/24 (37.5)	182/297 (61.3)
Nef RY11-1K		K - - - - - - - - - -	35/321 (10.9)	13/24 (54.2)	22/297 (7.4)
Nef RY11-1K10N		K - - - - - - - - N -	1/321 (0.3)	1/24 (4.2)	0/297 (0)
Nef RY11-10N		- - - - - - - - - N -	1/321 (0.3)	1/24 (4.2)	0/297 (0)
Nef RY11-11F		- - - - - - - - - - F	61/321 (19.0)	0/24 (0)	61/297 (20.5)
Pol AL10	Pol663-672	AQPDKSESEL	289/348 (83)	13/24 (54.2)	276/324 (85.2)
Pol AL10-10I		- - - - - - - - - I	53/348 (15.2)	7/24 (29.2)	46/324 (14.2)
Gag TL9	Gag180-188	TPQDLNTML	337/351 (96.0)	15/15 (100)	322/336 (95.8)
Gag TL9-3S7L		- - S - - - L - -	8/351 (2.3)	0/15 (0)	8/336 (2.4)
Gag TL9-3A7L		- - A - - - L - -	2/351 (0.6)	0/15 (0)	2/336 (0.6)
Gag NL11	Gag327–337	NPDCKTILKAL	334/341 (97.9)	14/14 (100)	320/327 (97.9)
Gag NL11-6I10S		- - - - - I - - - S-	3/341 (0.9)	0/15 (0)	3/327 (0.9)
Gag NL11-6L10S		- - - - - L- - - S-	2/341 (0.6)	0/15 (0)	2/327 (0.6)

**FIG 7:**
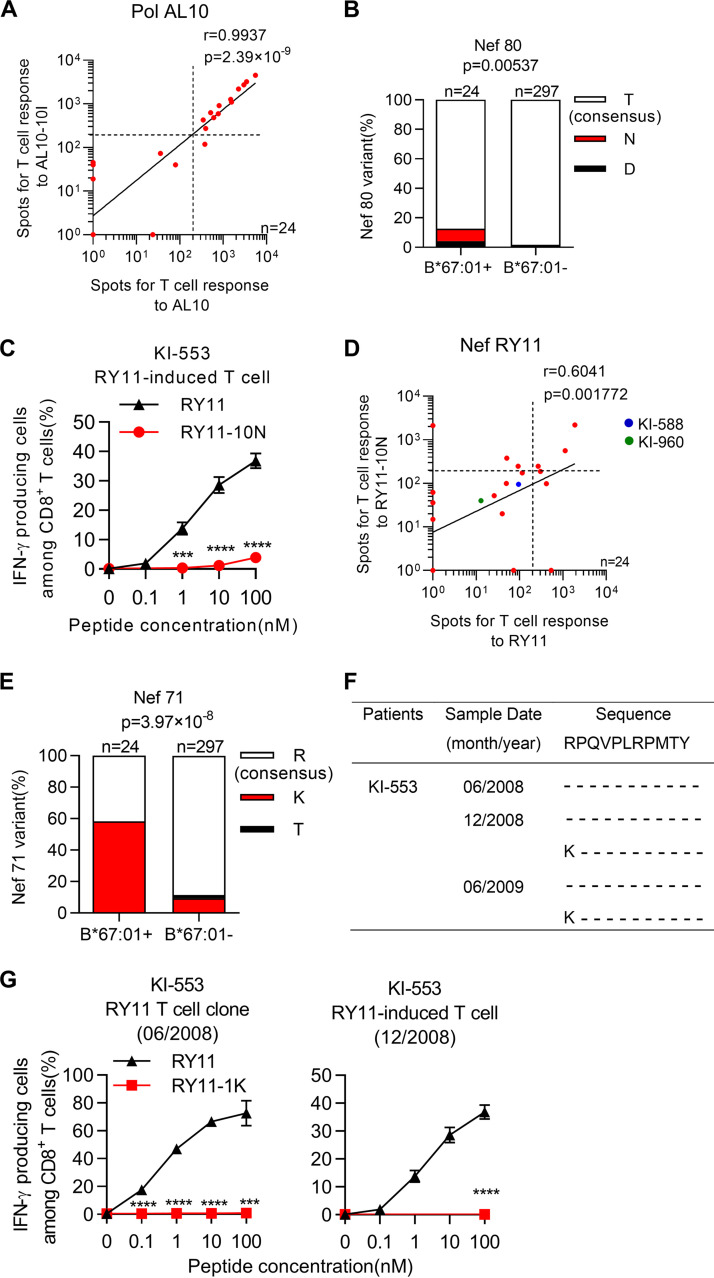
Effect of two mutations on HLA-B*67:01-restricted T-cell recognition. (A) Correlations of T-cell responses to the Pol AL10 epitope with those to Pol AL10-10I. T cell responses to the peptide at a concentration of 100 nM were measured by performing an *ex vivo* ELISpot assay in 24 treatment-naive HLA-B*67:01^+^ individuals. The dotted line at 200 spots/10^6^ CD8^+^ T cells indicates the threshold for a positive response. (B) The amino acid sequence at Nef80 was compared between 24 HLA-B*67:01^+^ and 297 HLA-B*67:01^–^ individuals. (C) Recognition of the RY11-10N mutant epitope by RY11-induced bulk T cells from an HLA-B*67:01^+^ individual, KI-553. T cell responses to the 721.221-B*67:01 cells pulsed with the RY11 or RY11-10N peptide from 0.1 to 100 nM were analyzed by performing the ICS assay. (D) Correlations of T-cell responses to the Nef RY11 epitope with those to Nef RY11-10N. (E) The amino acid sequence at Nef71 was compared between 24 HLA-B*67:01^+^ and 297 HLA-B*67:01^–^ individuals. (F) Longitudinal sequence analysis at Nef71 in an HLA-B*67:01^+^C*07:02^+^ individual (KI-553) infected with the HIV-1 subtype B virus. (G) Recognition of the RY11-1K mutant epitope by RY11-specific clones and RY11 peptide-induced bulk T cells generated from PBMCs collected from KI-553 in June 2008 (left) and December 2008 (right), respectively. T cell response to the 721.221-B*67:01 cells pulsed with the RY11 or RY11-1K peptide from 0.1 to 100 nM were analyzed by performing the ICS assay. Results are given as the mean with SD (*n* = 3; C and G). Associations of HLA-B*67:01 with the NefT80N and NefR71K mutations were statistically analyzed by using Fisher’s exact test (B and E). Statistical analysis was performed by the unpaired *t* test (C and G). ***, *P < *0.001; ****, *P < *0.0001.

Next, we analyzed T-cell responses to PolAL10 and PolAL10-10I peptides using the ELISpot assay. We found that 92.3% of the responders to the PolAL10 peptide had positive responses to the PolAL10-10I peptide, and the responses to the PolAL10-10I peptide positively correlated with the responses to the WT peptide (Fig. 7A). These findings indicate that the PolAL10-10I mutant epitope was cross-recognized by PolAL10-specific T cells. Thus, HLA-B*67:01-restricted T cells specific for two Gag and one Pol protective epitopes can recognize the majority of circulating viruses.

### Selection and accumulation of mutations in the NefRY11 epitope.

Three mutations, NefR71K, NefT80N, and NefY81F, were found in the NefRY11 epitope. NefT80N was found in only 2 of 24 HLA-B*67:01^+^ individuals (KI-588 and KI-960), whereas this mutation was not found in 297 HLA-B*67:01^–^ individuals (Table 3). Statistical analysis showed weak but significant association of NefT80N with HLA-B*67:01 (Fig. 7B), suggesting that this mutation may be selected by HLA-B*67:01-restricted T cells. Indeed, the NefRY11-specific T-cell line failed to recognize Nef RY11-10N (Fig. 7C). Analysis of T-cell responses to NefRY11 and NefRY11-10N in 24 HLA-B*67:01^+^ individuals demonstrated that three types of the specific T cells (Nef RY11-specific, NefRY11-10N-specific, and cross-reactive T-cells) were elicited in other HLA-B*67:01^+^ individuals (Fig. 7D), suggesting different T-cell responses to this mutant epitope among the HLA-B*67:01^+^ individuals.

The NefRY11-1K or NefRY11-1K10N mutant sequence was detected in 11.2% of 321 HIV-1 subtype B-infected individuals and in 58.3% of 24 HIV-1 subtype B-infected HLA-B*67:01^+^ individuals (Table 3). Statistical analysis demonstrated a strong HLA-B*67:01 association with NefR71K (*P = *3.97 × 10^−8^; Fig. 7E), suggesting that NefR71K is selected by HLA-B*67:01 and accumulated in the HLA-B*67:01^+^ individuals. We performed a longitudinal sequence analysis in an HLA-B*67:01^+^ individual (KI-553) whose longitudinal samples were available for this study. This individual was infected with the WT virus at the first sampling and then with a mixture of WT and NefR71K mutant viruses in December 2008 and June 2009 (Fig. 7F). We established an HLA-B*67:01-restricted RY11-specific T-cell clone from PBMC samples that were collected in June 2008, and RY11-specific bulk T cells from his PBMCs were collected in December 2008. These T cells failed to recognize 721.221-B*67:01 cells prepulsed with the RY11-1K mutant peptide (Fig. 7G), indicating that the NefR71K mutant virus was selected by the HLA-B*67:01-restricted RY11-specific T cells.

**FIG 8:**
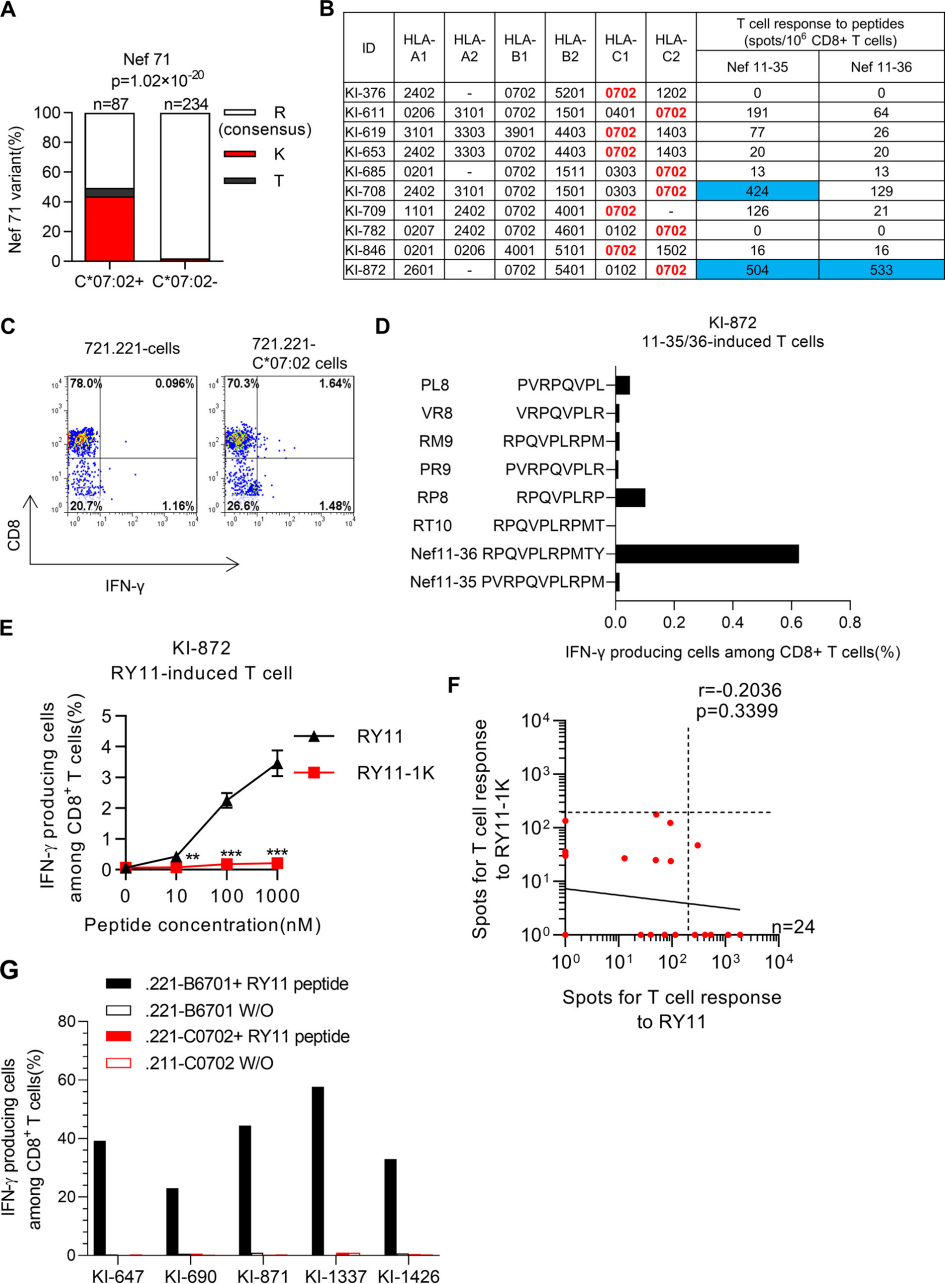
Selection and accumulation of the NefR71K mutation by HLA-C*07:02-restricted Nef RY11-specific T cells. (A) The amino acid sequence at Nef71 was compared between 87 HLA-C*07:02^+^ and 234 HLA-C*07:02^–^ individuals. The association of HLA-C*07:02 with the NefR71K mutation was statistically analyzed by using Fisher’s exact test. (B) Responses of PBMCs from 10 HIV-1-infected HLA-B*67:01-C*07:02^+^ individuals to two overlapping 11-mer peptides, Nef11-35/36, covering Nef71 at a concentration of 1 μM were analyzed by performing an *ex vivo* IFN-γ ELISpot assay. The blue shading indicates the positive responses (>200 spots) in the ELISpot assay. (C) HLA-restriction of Nef11-35/36-induced bulk T cells derived from KI-872. Responses of Nef11-35/36-induced bulk T cells to the 721.221 or 721.221-C*07:02 cells pulsed with the Nef11-35/36 peptide at 100 nM were analyzed by performing the ICS assay. The results of flow cytometric analysis are shown. (D) Identification of the HLA-C*07:02-restricted optimal epitope peptide. Responses of the Nef11-35/36-induced bulk T cells to the 721.221-C*07:02 cells pulsed with Nef11-35/36 peptides or those with truncated ones were analyzed by performing the ICS assay. (E) Recognition of the RY11-1K mutant epitope by RY11 peptide-induced bulk T cells from an HLA-B*67:01-C*07:02^+^ individual, KI-872. T cell response to the 721.221-C*07:02 cells pulsed with the RY11 or RY11-1K peptide from 0.1 to 100 nM were analyzed by using the ICS assay. Results are given as the mean with SD (*n* = 3). Statistical analysis was performed by the unpaired *t* test. **, *P* < 0.01; ***, *P* < 0.001. (F) Correlations of T-cell responses to the NefRY11 epitope with those to Nef RY11-1K. T-cell responses to the peptide at a concentration of 100 nM were measured by performing the *ex vivo* ELISpot assay in 24 treatment-naive HLA-B*67:01^+^ individuals. T-cell responses to the WT peptide and the mutant one in each individual are presented. The dotted line at 200 spots/10^6^ CD8^+^ T cells indicates the threshold for a positive response. (G) HLA-restriction of RY11 peptide-induced bulk T cells from 5 responders. Responses of RY11-induced bulk T cells to the 721.221-B*67:01 or 721.221-C*07:02 cells pulsed with the RY11 peptide at 100 nM were analyzed by performing the ICS assay.

Previously, it has been shown that NefR71K is an HLA-C*07:02-associated mutation in an HIV-1 subtype B-infected Japanese population (35). To confirm this result, we reanalyzed the HLA-C*07:02 association with NefR71K in this cohort. The results showed a strong association of NefR71K with HLA-C*07:02 (*P = *1.02 × 10^−20^; Fig. 8A), suggesting that this mutation is also selected by HLA-C*07:02-restricted T cells. Because the HLA-C*07:02-restricted epitope covering Nef71 has not been reported, we sought to identify HLA-C*07:02-restricted epitopes including Nef71. We first selected 10 HLA-C*07:02^+^B*67:01^–^ responders to the Nef4 peptide cocktail, including 11-mer peptides covering Nef71 (Nef11-35 and Nef11-36), and then tested the responses of PBMCs from these individuals to Nef11-35 and Nef11-36. Responses to these two peptides were detected in individual KI-872, while a response to only Nef11-35 was found in individual KI-708 (Fig. 8B). We generated specific T cells by stimulating PBMCs from individual KI-872 with Nef11-35/Nef11-36 peptides and then tested the recognition of these 11-mer peptides by the bulk T cells by performing the ICS assay using 721.221 cells expressing HLA-C*07:02 (721.221-C*07:02 cells). Nef11-35/Nef11-36-induced T cells derived from individual KI-872 exhibited HLA-C*07:02-restricted Nef11-35/Nef11-36-specific T cell responses (Fig. 8C). To determine the optimal peptide of the HLA-C*07:02-restricted T cells, we investigated the recognition of the truncated peptides by HLA-C*07:02-restricted bulk T cells and found that RY11 is an optimal HLA-C*07:02-restricted epitope (Fig. 8D). Further analysis using RY11-specific HLA-C*07:02-restricted T cells demonstrated that these T cells failed to recognize 721.221-C*07:02 cells prepulsed with the NefRY11-1K mutant peptide (Fig. 8E), suggesting that the HLA-C*07:02-restricted T cells can select the NefR71K mutant virus.

Next, we investigated T-cell responses to NefRY11-1K by performing the *ex vivo* ELISpot assay in 24 HLA-B*67:01^+^ individuals and found that T-cell responses to NefRY11-1K were not detected in any of the individuals tested, although NefRY11-specific T cells were found in 6 individuals (Fig. 8F). Taken together, these results suggest that NefRY11-1K is an escape mutant epitope from RY11-specific T cells. Because all six responders to NefRY11 had both HLA-B*67:01 and HLA-C*07:02, it is not clear whether the response to RY11 results from an HLA-B*67:01-restricted or HLA-C*07:02-restricted T-cell response. To elucidate the HLA-restriction of these T-cell responses, we analyzed the HLA-restriction of NefRY11 peptide-induced bulk T cells from five individuals whose PBMCs were available for this analysis. All these bulk T cells recognized 721.221-B*67:01 cells prepulsed with the RY11 peptide, but not 721.221-C*07:02 cells prepulsed with the same peptide, indicating that the T-cell responses to the RY11 peptide are restricted by HLA-B*67:01 (Fig. 8G). These results imply that NefR71K may be predominantly selected by the HLA-B*67:01-restricted T cells in HLA-C*07:02^+^B*67:01^+^ individuals.

## DISCUSSION

Although previous studies have suggested an advantage of rare HLA alleles against HIV-1 disease progression (5, 36), the effects of rare HLA alleles on the disease progression and its mechanism are still unclear. A previous study on HIV-1-infected Japanese individuals showed that HLA-B*67:01^+^ individuals exhibited the lowest pVL and highest CD4 counts among HIV-1 clade B-infected Japanese individuals (8). The prevalence of HLA-B*67:01 in Japan is approximately 1.2%, whereas that of HLA-B*57 in Caucasian and Black African populations is 2.8% to 8% (37–43). Thus, the rare HLA advantage in HIV-1 infection was found in the Japanese individuals harboring HLA-B*67:01. Because a previous study identified only Gag epitopes presented by HLA-B*67:01 (24), the mechanism of the protective effect of HLA-B*67:01 remains unclear. In the present study, we identified six novel HLA-B*67:01-restricted epitopes and demonstrated that four novel epitopes, NefRY11, NefRM9, NefRL9, and PolAL10, were immunodominant in addition to two previously identified Gag epitopes. Further analysis of T-cells specific for these epitopes showed that responders to NefRY11, PolAL10, GagTL9, or GagNL11 had higher CD4 counts and/or lower pVL than nonresponders. The breadths and total magnitude of T-cell responses to NefRY11, PolAL10, GagTL9, and GagNL11 showed a strong positive correlation with the CD4 count and an inverse correlation with the pVL. Thus, the present study demonstrated that these HLA-B*67:01-restricted T-cells had a strong ability to suppress HIV-1 replication in HIV-1-infected individuals.

NefRY11 and PolAL10 are less conserved than the two Gag epitopes among the four HLA-B*67:01 protective epitopes. Isoleucine at the C terminus of PolAL10 (PolL672I) was most frequently detected among these substitutions. However, PolL672I was not an HLA-B*67:01-associated mutation, suggesting that this is not an escape mutation from PolAL10-specific T cells. Indeed, this mutant peptide was cross-recognized by PolAL10-specific T cells. These findings indicate that PolAL10-specific T cells can recognize most of the circulating viruses. NefY81F mutation in NefRY11 was detected in 20% of 321 individuals but not in any of the HLA-B*67:01^+^ individuals, indicating that it is not an HLA-B*67:01-associated mutation. On one hand, a previous study demonstrated that NefY81F mutation is associated with HLA-B*35:01 (35), suggesting that this mutation was selected by HLA-B*35:01-restricted NefRY11-specific CD8^+^ T cells. On the other hand, the NefR71K mutant associated with HLA-B*67:01. NefRY11-specific T cells failed to recognize the RY11-1K mutant epitope, and the T-cell response to the RY11-1K mutant peptide was not detected in any of the HLA-B*67:01^+^ individuals, including those who showed the T-cell response to RY11 peptides. These findings strongly suggest that NefR71K is an escape mutation selected by HLA-B*67:01-restricted RY11-specific CD8^+^ T cells. The current study also confirmed the association of NefR71K with HLA-C*07:02 and demonstrated that HLA-C*07:02-restricted RY11-specific CD8^+^ T cells failed to recognize the RY11-1K mutant epitope. These findings suggest that both types of T cells may contribute to selection and accumulation of this mutation. However, the HLA-C*07:02-restricted NefRY11-specific T cells were not found in the five HLA-B*67:01^+^HLA-C*07:02^+^ responders to NefRY11 (Fig. 8G). These findings imply that the HLA-B*67:01-restricted RY11-specific T cells contribute to selection of this mutant in the HLA-B*67:01^+^HLA-C*07:02^+^ individuals, while HLA-C*07:02-restricted NefRY11-specific T cells may select this mutation in the HLA-B*67:01-C*07:02^+^ ones. NefR71K was found in 14 of 24 HLA-B*67:01^+^ individuals (Table 3), and the T-cells response to the NefRY11 epitope was found in only 3 of 14 individuals infected with the NefR71K mutant (data not shown). These findings support the idea that selection and accumulation of NefR71K by HLA-C*07:02-restricted RY11-specific T cells may negatively affect the induction of HLA-B*67:01-restricted RY11-specific T cells if this mutant is transmitted to the HLA-B*67:01^+^ individuals.

It is assumed that NefRY11-specific T cells failed to suppress the replication of the Nef71K mutant virus in HLA-B*67:01^+^ individuals infected with this mutant virus because NefRY11-specific T cells failed to recognize the RY11-1K mutant epitope. We found that three out of five responders to NefRY11 were infected with the Nef71K mutant virus, whereas the other two responders were infected with the WT virus. Responders harboring the mutant virus showed a trend for higher pVL and lower CD4 counts than those harboring the WT virus (median pVL, 61,000 versus 4,850; median CD4 count, 496 versus 899). Although a small number of HLA-B*67:01^+^ responders were analyzed, this result implied that NefRY11-specific T cells failed to suppress the replication of the Nef71K mutant virus in HLA-B*67:01^+^ individuals infected with the mutant virus. This finding together with the result that RY11-1K mutant-specific T cells were not induced in individuals infected with the Nef71K mutant virus (Fig. 8F) suggest that the accumulation of this mutant virus in the population negatively affected HIV-1 control by NefRY11-specific T cells in HLA-B*67:01^+^ individuals.

Our previous study analyzed 504 treatment-naive individuals, including 11 HLA-B*67:01^+^ individuals who were recruited from 2000 to 2010, and showed that clinical outcomes in the HLA-B*67:01^+^ individuals were significantly better than those in HLA-B*67:01^–^ individuals (8). However, this protective effect of HLA-B*67:01 became weaker in the current study of 838 treatment-naive individuals, including 24 HLA-B*67:01^+^ individuals who were recruited from 2007 to 2019. Among the 12 HLA-B*67:01^+^ individuals recruited from 2000 to 2010, 5 and 7 were infected with the Nef17K mutant or WT viruses, respectively, whereas 9 and 3 of 12 HLA-B*67:01^+^ individuals recruited from 2011 to 2019 were infected with the Nef71K mutant or WT viruses, respectively (data not shown). From these findings, we speculated that the accumulation of Nef71K negatively weakened the protective effect of HLA-B*67:01 in Japan over the past decade.

Previous studies have demonstrated that polyfunctional and broad CD8^+^ T-cell responses were induced by an HIV-1 conserved vaccine and that CD8^+^ T cells specific for the conserved region contributed to suppression of HIV-1 replication in HIV-1 infection (34, 44–48). Here, we identified that the CD8^+^ T cells specific for four HLA-B*67:01-restricted epitopes were significantly associated with good clinical outcomes and that the majority of these protective epitopes were conserved and had a strong effect on suppression of HIV-1 replication. The present study provided additional evidence supporting the concept that CD8^+^ T cells specific for the conserved region have a strong ability to suppress HIV-1 replication. Thus, CD8^+^ T cells targeting the conserved regions are preferable candidates of effector T cells for an AIDS vaccine (49–54) and the HIV-1 cure treatment (55).

GagTL9 is known to be an HLA-B*81:01-restricted epitope in HIV-1 subtype C infection (56, 57). HLA-B*81:01 is associated with slow progression to AIDS in African populations infected with HIV-1 subtype C strains and the second strongest protective allele in southern Africa (6, 30, 58–60). Previous studies have shown that the magnitude of T-cell responses to the GagTL9 epitope was associated with good clinical outcomes in HLA-B*81:01^+^ black African individuals (56, 61). Thus, GagTL9 is a protective T-cell epitope presented by both HLA-B*67:01 and HLA-B*81:01. Furthermore, previous studies have shown HLA-B*81:01-associated escape mutations (GagQ182S and GagT186S) within the GagTL9 epitope in chronically HIV-1 subtype C-infected individuals (62, 63) and have shown that these mutations were found in 32% of HIV-1 subtype C-infected HLA-B*81:01^+^ individuals (62). The emergence of these escape mutants at a late phase of the infection was associated with increased viremia (59). In contrast, the current study in Japan showed that GagTL9 is highly conserved among the circulating viruses and that the GagQ182S/A and GagT186L mutations were found in only 2.9% of the circulating viruses, but they were not detected in the HLA-B*67:01^+^ individuals. Thus, the accumulation of these escape mutant viruses is only detected in black African individuals, even though protective GagTL9-specific T cells are elicited in both Japan and Africa. Because these mutations are associated with HLA-B*81:01 in Africa, we assume that GagTL9-specific HLA-B*81:01-restricted T cells can select these mutant viruses. A previous study demonstrated that HLA-B*67:01-restricted T cells cannot recognize GagTL9-3S-7L/TL9-3A-7L mutant peptides (64), implying that these T cells can select these mutant viruses in Japan. However, the reason for the accumulation of these mutations in Africa but not in Japan remains unknown.

HLA-B*67:01 has been reported as one of the HLA alleles associated with autoimmune diseases such as Takayasu arteritis and relapsing polychondritis (65–68). HLA-B57 and HLA-B*27 are associated with drug hypersensitivity (69, 70) and autoimmune diseases such as ankylosing spondylitis (71, 72), respectively, while HLA-B*52:01, which is a protective allele in HIV-1-infected Japanese individuals, is also associated with Takayasu arteritis (65, 66, 68, 73–75) and ulcerative colitis (65, 68, 76). These findings suggest that strong immune responses elicited by these HLA molecules contribute to not only the control of HIV-1 but also the onset of autoimmune diseases and hypersensitivity. It is interesting that these HLA alleles have different effects on infectious diseases and autoimmune diseases.

In the present study, we identified four protective HLA-B*67:01-restricted epitopes and demonstrated that three of them were conserved among circulating HIV-1 viruses. CD8^+^ T-cells specific for these epitopes were effectively and stably elicited in the majority of HIV-1-infected HLA-B*67:01^+^ individuals. Thus, we demonstrated that HLA-B*67:01 has a strong effect on HIV-1 suppression via immune control by HLA-B*67:01-restricited T cells specific for conserved epitopes and that preadapted mutation affected the recognition of Nef-specific T cells. In the current study, we elucidated part of the mechanism of the rare HLA allele advantage against HIV-1 infection.

## MATERIALS AND METHODS

### Subjects.

All treatment-naive Japanese individuals chronically infected with HIV-1 subtype B were recruited between 2007 and 2019 from the National Center for Global Health and Medicine, Japan. This study was approved by the ethics committees of Kumamoto University (RINRI-1340 and GENOME-342) and the National Center for Global Health and Medicine (NCGM-A-000172-01). Informed consent was obtained from all individuals in accordance with the Declaration of Helsinki. Peripheral blood mononuclear cells (PBMCs) were separated from whole blood. The HLA type of HIV-infected individuals was determined by standard sequence-based genotyping. The pVLs of the individuals were measured using the Cobas TaqMan HIV-1 real-time PCR version 2.0 assay (Roche Diagnostics, NJ, USA).

### Peptides.

We previously designed overlapping peptides consisting of 11-mer amino acids spanning consensus sequences of Gag, Pol, and Nef of HIV-1 clade B (77). Each 11-mer peptide was overlapped by 9 amino acids. These 11-mer peptides and truncated peptides were synthesized by an automated multiple peptide synthesizer and purified by high-performance liquid chromatography (HPLC). The purity was examined by HPLC and mass spectrometry. Peptides with more than 90% purity were used.

### IFN-γ ELISpot assay.

An *ex vivo* IFN-γ ELISpot assay was performed as previously described (24, 48). The number of spots of a T-cell response to each peptide was finally calculated by subtracting the number of spots in the wells without peptides from that in the wells with peptides. The mean value plus 3 standard deviations of spot number for the peptides among 13 HIV-1-naive individuals was 162 spots/10^6^ CD8^+^ T cells (24, 48). Therefore, we defined >200 spots/10^6^ CD8^+^ T cells as a positive response.

### Cell lines.

721.221 cells expressing CD4 molecules and HLA-B*67:01 (721.221-B*67:01) or HLA-C*07:02 (721.221-C*07:02) were previously generated (24). These cells were maintained in RPMI 1640 medium (Invitrogen) containing 5% fetal calf serum (FCS, R5) and 0.15 mg/mL hygromycin B or 0.2 mg/mL neomycin.

### Induction of HIV-1-specific T cells from HIV-1-infected individuals.

PBMCs from individuals KI-699, KI-553, and KI-475 were incubated with a 1-μM 11-mer peptide cocktail of Nef4 or Nef7, Nef4, and Pol4 or Pol37, respectively, and cultured for 14 days to induce peptide-specific T cells. To establish HIV-1 epitope-specific bulk T cells, PBMCs were stimulated with a specific epitope peptide at 100 nM. After 2 to 3 weeks in culture, epitope-specific bulk T cells were analyzed by performing the intracellular cytokine staining (ICS) assay. The NefRY11 T-cell clone was generated from the RY11-specific bulk T cells by the limiting dilution assay in a 96-U plate, using 200 μL of cloning mixture of 2 × 10^5^ irradiated allogeneic PBMCs from healthy donors, 2 × 10^4^ irradiated 721.221-B*67:01 cells, and 100 nM RY11 peptide in RPMI 1640 containing FCS, 200 U/mL rIL-2, and 2.5% phytohemagglutinin (PHA). After 2 to 3 weeks in culture, the NefRY11-specific CD8^+^ T-cell clone was used in the ICS assay.

### Preparation of HIV-1 clones.

The full-length HIV-1 pNL43 derivative in which the *Nef* gene was completely replaced with SF2 *NEF* (pNL43_SF2Nef_) was previously generated (78, 79). 293T cells were transfected with the construct, and the infectious HIV-1 virions released into the medium were collected 72 h later.

### Intracellular cytokine staining (ICS) assay.

721.221 cells prepulsed with HIV-1 epitope peptide or infected with HIV-1 virus, or CD4^+^ T cells infected with HIV-1 virus were cocultured with HIV-1-specific bulk-cultured T cells or a T-cell clone in a 96-well plate for 2 h at 37°C. Brefeldin A (10 μg/mL) was then added, and the cells were incubated for another 4 h at 37°C. Next, the cells were fixed with 4% paraformaldehyde and incubated in permeabilization buffer (0.1% saponin, 10% FBS-phosphate-buffered saline [PBS]), after which they were stained with allophycocyanin (APC)-conjugated anti-CD8 monoclonal antibody (MAb; Dako, Denmark), followed by fluorescein isothiocyanate (FITC)-conjugated anti-IFN-γ MAb (BD Biosciences). The percentage of IFN-γ-producing cells among the CD8^+^ T-cell population was determined by FACS Canto II.

### Identification of the HIV-1 epitope sequence.

Determination of the epitope sequence was performed as previously described (35). Briefly, viral RNA was extracted from plasma samples using a QIAamp MinElute virus spin kit (Qiagen). cDNA was synthesized from the RNA using the SuperScript III first-strand synthesis system for reverse transcription-PCR (RT-PCR) and random hexamers (Invitrogen). The *Nef*, *Gag*, and *Pol* regions were amplified by nested PCR using *Taq* DNA polymerase (Promega). All sequences were determined using the BigDye Terminator version 3.1 cycle sequencing kit (Applied Biosystems) and ABI 3500 genetic analyzer (Applied Biosystems). The epitope sequence data of NefRY11 from 321 chronically HIV-1 subtype B-infected, treatment-naive Japanese individuals and the epitope sequence data of PolAL10 from 348 individuals were analyzed after excluding individuals with a mixture of amino acid sequences from previously analyzed data (35) and adding new data from nine HLA-B*67:01 individuals. The GagTL9 and GagNL11 sequence data were previously identified from 351 chronically HIV-1 subtype B-infected individuals (24).

### Data availability.

The sequences have been deposited in DDBJ/EMBL/GenBank under accession numbers AB873205 to AB873601 (Gag), AB873908 to AB874270 and LC730810 to LC730827 (Pol), and AB873602 to AB873907 and LC730828 to LC730844 (Nef).

### Statistical analysis.

The frequency of individuals harboring the mutants between HLA^+^ and HLA^–^ ones was statistically analyzed using Fisher’s exact test. Groups were compared by performing the unpaired *t* test or two-tailed Mann-Whitney U tests. A *P* value of* <*0.05 was considered significant. Multiple tests were performed using the *q* value, a measure of significance in terms of the false-discovery rate. A significance threshold for *q* of <0.1 was used.
